# Supplementary material for: Analysis of chi angle distributions in free amino acids via multiplet fitting of proton scalar couplings
Source: Magn Reson (Gott). 2024 Aug 19;5(2):103–20. doi: 10.5194/mr-5-103-2024 (PMC11570886; doi:10.5194/mr-5-103-2024)
Supplement: The supplement related to this article is available online at: https://doi.org/10.5194/mr-5-103-2024-supplement. [file mr-5-103-2024-supplement.zip › Syed2024/data/fit1d_fitnmr_output.tar.gz!/fit1d/tyr/500/bmse000051_simulation_1_nmredata/1H/resonances_1d.pdf]

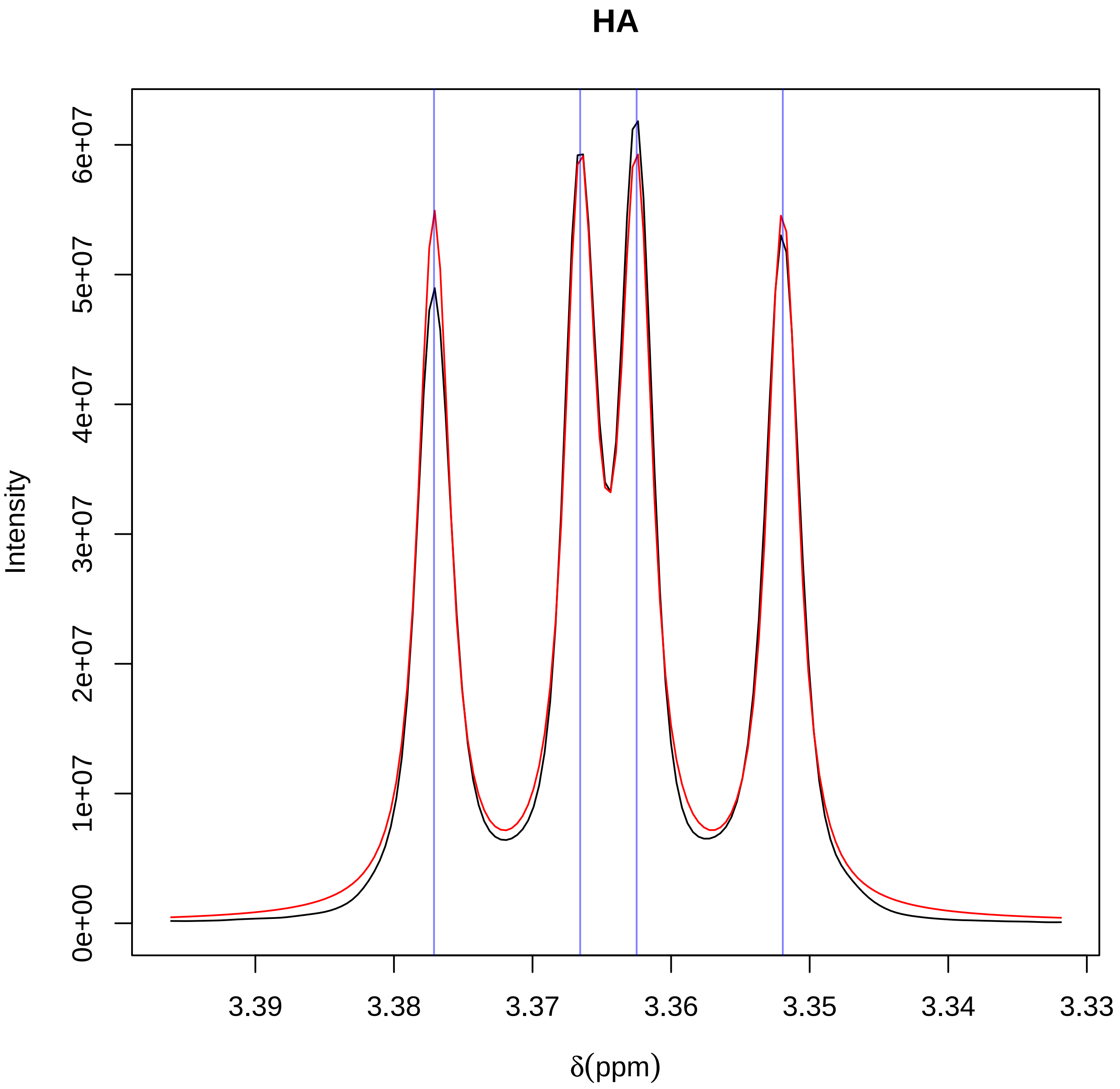
HA
Intensity
0e+00
1e+07
2e+07
3e+07
4e+07
5e+07
6e+07
3.39
3.38
3.37
3.36
3.35
3.34
3.33
δ(ppm)

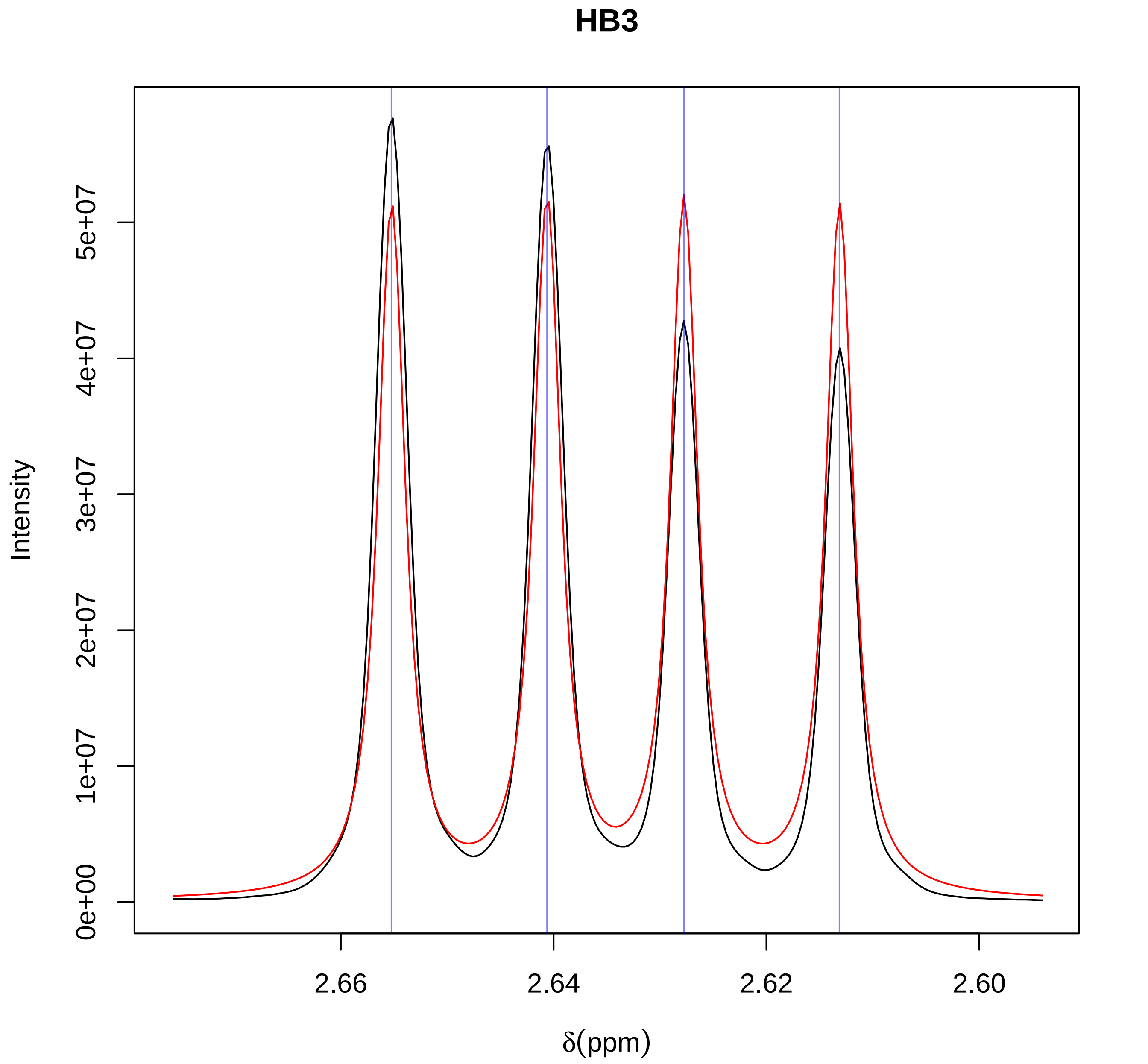

HB3
Intensity
0e+00
1e+07
2e+07
3e+07
4e+07
5e+07
2.66
2.64
2.62
2.60
δ(ppm)

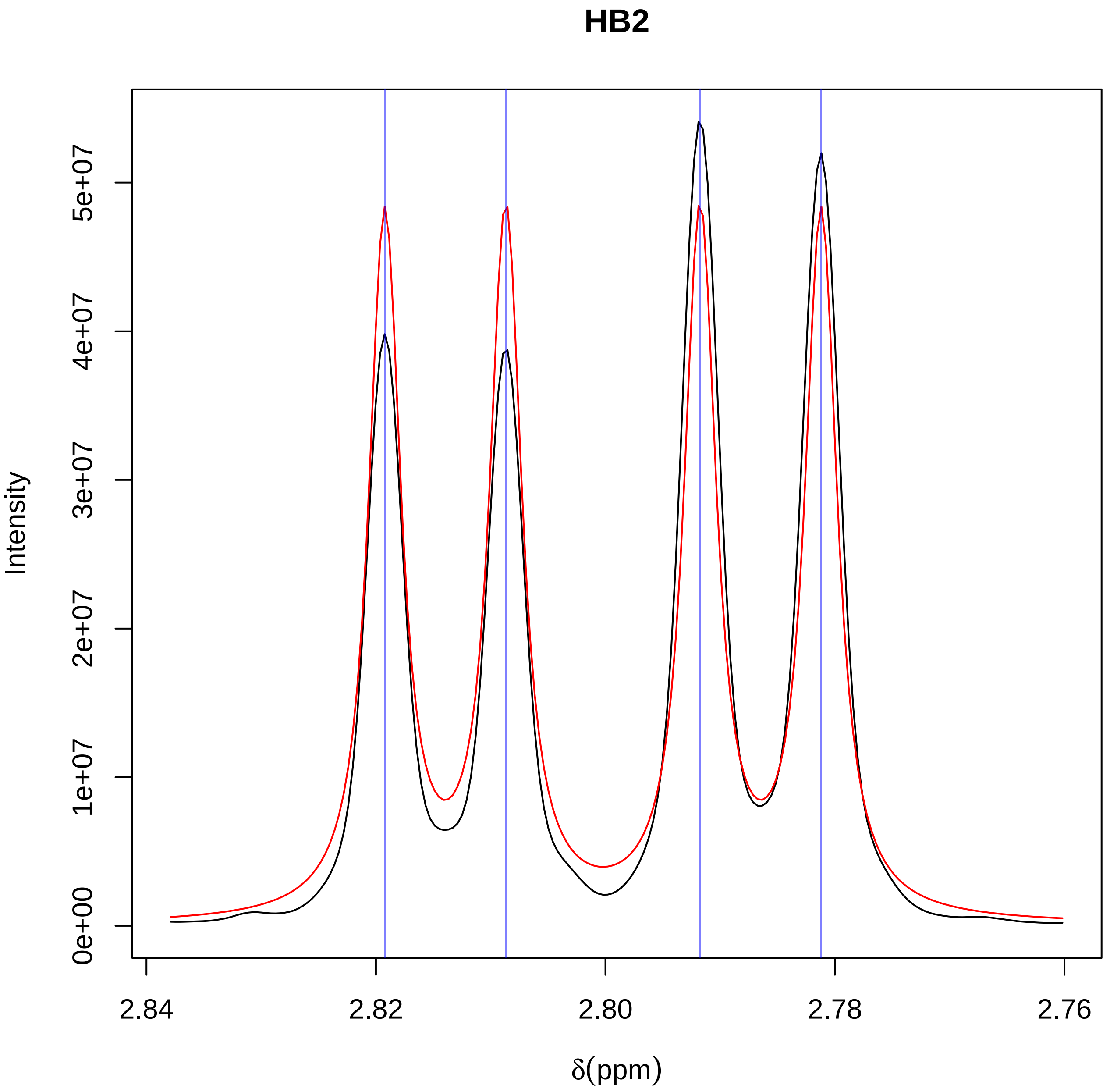

HB2
Intensity
0e+00
1e+07
2e+07
3e+07
4e+07
5e+07
2.84
2.82
2.80
2.78
2.76
δ(ppm)

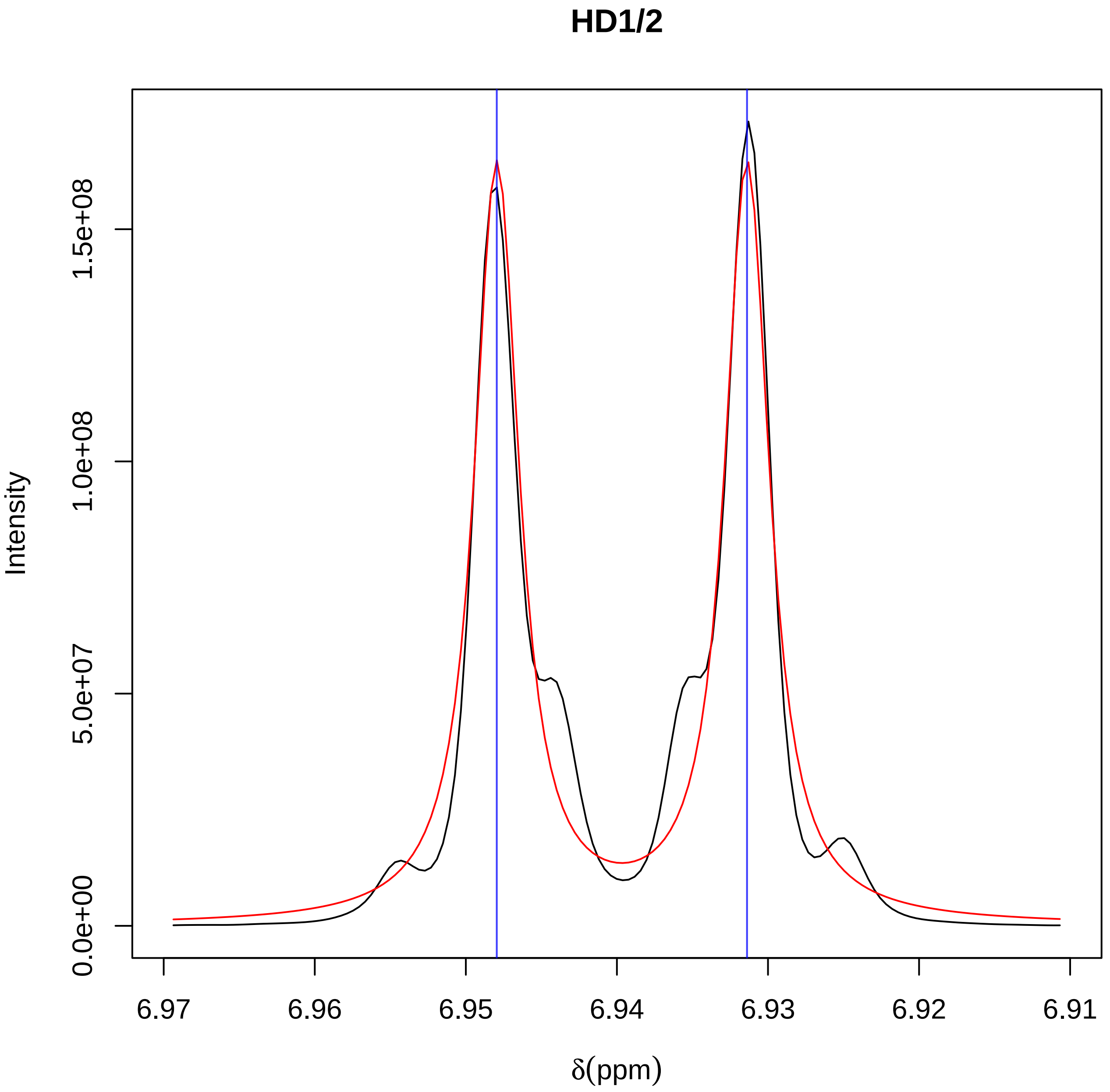
HD1/2
Intensity
0.0e+00
5.0e+07
1.0e+08
1.5e+08
6.97
6.96
6.95
6.94
6.93
6.92
6.91
δ(ppm)

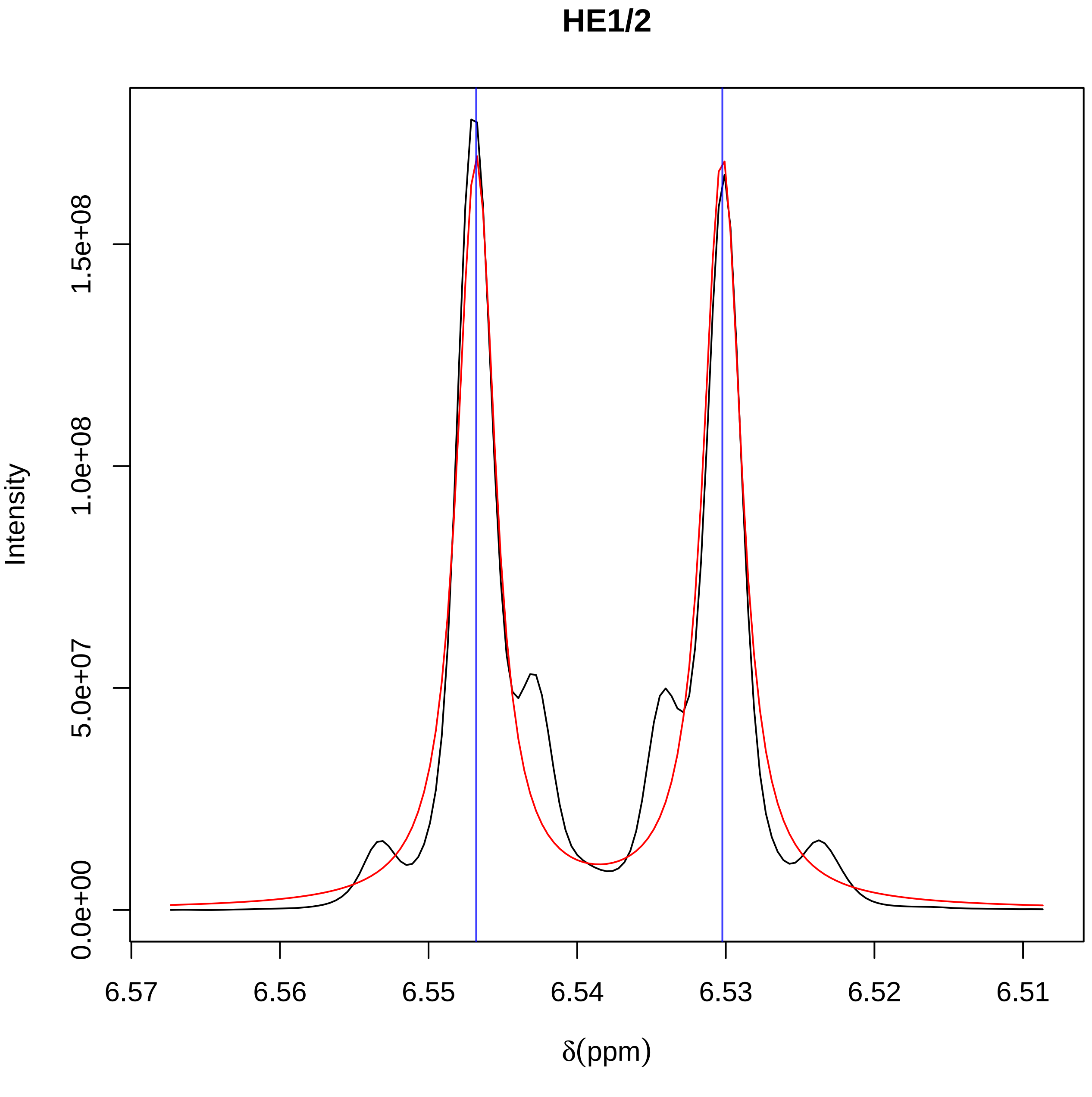

HE1/2
Intensity
0.0e+00
5.0e+07
1.0e+08
1.5e+08
6.57
6.56
6.55
6.54
6.53
6.52
6.51
δ(ppm)
